# Network biomarkers of Alzheimer’s disease risk derived from joint volume and texture covariance patterns in mouse models

**DOI:** 10.1371/journal.pone.0327118

**Published:** 2025-08-12

**Authors:** Eric W. Bridgeford, Jaewon Chung, Robert J. Anderson, Ali Mahzarnia, Jacques A. Stout, Hae Sol Moon, Zay Yar Han, Joshua T. Vogelstein, Alexandra Badea

**Affiliations:** 1 Department of Biomedical Engineering, Johns Hopkins University, Baltimore, Maryland, United States of America; 2 Stanford University, Stanford, California, United States of America; 3 Radiology Department, Duke University Medical School, Durham, North Carolina, United States of America; 4 Brain Imaging and Analysis Center, Duke University Medical School, Duke University Medical School, Durham, North Carolina, United States of America; 5 Biomedical Engineering, Pratt School of Engineering, Duke University, Durham, North Carolina, United States of America; 6 Neurology Department, Duke University Medical School, Duke University Medical School, Durham, North Carolina, United States of America; University of Thessaly Faculty of Medicine: Panepistemio Thessalias Tmema Iatrikes, GREECE

## Abstract

Alzheimer’s disease (AD) lacks effective cures and is typically detected after substantial pathological changes have occurred, making intervention challenging. Alzheimer’s disease (AD) intervention requires early detection of risk factors and understanding their complex interactions before substantial pathological changes manifest. Current research often examines individual risk factors in isolation, limiting our understanding of their combined effects. We present a novel multivariate analytical framework to simultaneously assess multiple AD risk factors using mouse models expressing human ApoE alleles. Our methodological innovation lies in combining high-resolution magnetic resonance diffusion imaging with a comprehensive multifactorial analysis that integrates genotype, age, sex, diet, and immunity as interacting variables. This approach enables the simultaneous examination of regional brain volume and fractional anisotropy changes across multiple risk factors, providing a more holistic view than traditional univariate analyses. Our proposed method effectively identified how these factors converge on specific brain regions – with genotype influencing the caudate putamen, pons, cingulate cortex, and cerebellum; sex affecting the amygdala and piriform cortex; and immune status impacting association cortices and cerebellar nuclei. Importantly, our integrated approach revealed factor interactions that would remain undetected in single-variable studies, particularly in the amygdala, thalamus, and pons. While many findings align with previous research, our multidimensional framework offers a methodological advancement for studying AD risk factors by modeling their combined effects rather than isolated impacts. This approach creates a template for future studies to investigate mechanisms underlying coordinated changes in brain structure through network analyses of gene expression, metabolism, and structural pathways involved in neurodegeneration.

## 1 Introduction

Alzheimer’s disease (AD) is the most common type of dementia and is estimated to affect more than 5 million U.S. citizens and more than 25 million people worldwide. The risk of Alzheimer’s disease (AD) is complex and multifactorial, resulting in multiple pathologies, and is influenced by factors including genetic predispositions [1], environmental factors [2], lifestyle [3], and most importantly age [4]. Therefore, identifying and understanding which regions of the brain are highly vulnerable, that is, subject to significant changes, and how different risk factors contribute to this vulnerability, is important to understand, bearing the potential to open new therapeutic targets and opens new horizons for AD prevention [5].

Our scientific premise is based on the role of alleles of the apolipoprotein E (ApoE) gene in aging and Alzheimer’s disease. ApoE is a critical gene involved in lipid metabolism and neuronal repair processes, with its variants: ApoE2, ApoE3, and ApoE4 playing distinct roles in neurodegenerative diseases [6,7]. The most studied among these, ApoE4, is known for its strong association with an increased risk of Alzheimer’s disease. In contrast, ApoE2 is considered neuroprotective, while ApoE3 is neutral. This genetic variability offers a unique opportunity to explore the connection between genetic profiles and brain structure, particularly in relation to neurodegenerative diseases. Mouse models with human targeted replacement ApoE alleles allow for understanding the mechanisms behind early alterations in such association at prodromal stages.

Recent research in AD is increasingly focused on understanding the relationship between brain volume variations, also known as structural covariance/correlation. A notable study demonstrated that single nucleotide polymorphisms (SNPs) susceptible to Alzheimer’s disease are closely related to changes in grey matter volume and cognitive outcomes [8]. This research utilized magnetic resonance imaging to construct grey matter structural covariance networks (SCNs) in patients with Alzheimer’s disease. The study assessed the effects of various genotypes loci on cognitive outcomes, revealing that specific genetic variations, including the ApoE4 allele, interact with or independently affect cognitive outcomes. Other studies similarly support the fact that there is a relationship between AD progression and changes in SCNs [9,10]. However, these methods exhibit limitations. The main limitation is that these methods construct a single SCN per group, which can lead to a small sample size when trying to find brain regions that are changing, rather than a single correlation between pairs of regions. Performing hypothesis tests on individual elements of a correlation matrix, whose data are highly correlated, can result in spurious conclusions if the interactions between regions are not decoupled.

**Fig 1:**
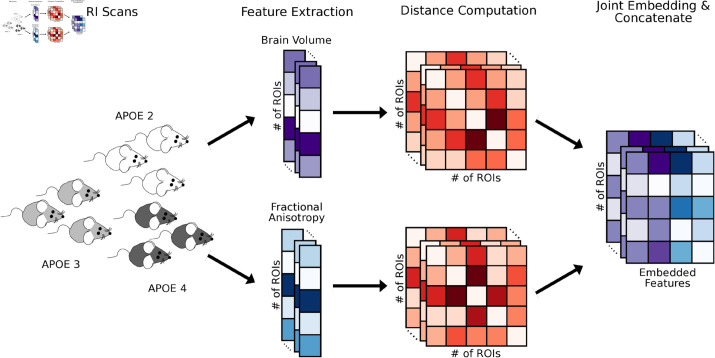
An illustration of the preprocessing pipeline. MRI scans from the three genotypes of mice, ApoE2, ApoE3, and ApoE4 are processed to estimate brain volume and fractional anisotopy data per ROI. Each data modality is then transformed into distance matrices, which are jointly embedded. Resulting embedded features are concatenated for subsequent analysis.

To address these issues, we propose a method for obtaining individualized representations of structural covariance, leveraging recent advances in structural brain imaging [11] with *K*-sample and multi-way hypothesis testing frameworks [12] to identify brain regions that are highly vulnerable, or susceptible to significant change. We first compute absolute differences of features between all pairs of brain regions, resulting in a network per subject. We focus on studying the brain volume derived from structural MRI, and fractional anisotropy derived from diffusion MRI. Given these networks, we obtain a low-rank representation for each brain region per subject by jointly modeling the networks. These data preprocessing steps are shown in Fig 1. We then leverage distance correlation to perform multi-way tests that include factors such as age, sex, genotypes, and diet.

We used magnetic resonance microscopy [13] to derive quantitative metrics reflective of brain microstructural integrity in mouse models with different risk actors for AD (age, ApoE genotype, sex, diet and immunity). Specifically, we employed diffusion imaging, since this can reveal multiple markers for both volume and microstructural changes [14]. Our results reveal significant influence of genetic risk factors, particularly in differentiating between amygdala network-associated and non-associated ApoE allelic populations. While alone age, sex, and diet emerge as marginal factors, their significance escalates when considering the broader ApoE allelic set. These insights not only improve our understanding of AD’s complexity but also signify a pivotal step towards stratified, personalized, and effective clinical interventions.

## 2 Materials and methods

### 2.1 Animals

We have used mice expressing the human apolipoprotein E (ApoE) alelles, critically involved in lipid metabolism and neuronal repair processes [15], homozygous for its variants–ApoE2/2 (referred to in the text as ApoE2), ApoE3/3 (ApoE3), or ApoE4/4 (ApoE4)–because of their distinct roles in neurodegenerative diseases. ApoE3 is the allele found in a majority of the population. The most studied among these, ApoE4, is known for its strong association with an increased genetic risk of Alzheimer’s disease. In contrast, ApoE2 is considered neuroprotective [16]. All mice have the human version of the ApoE allele, and some express the mouse nitric oxide synthase gene (Nos2) while others express the human nitric oxyde synthase 2 (NOS2) gene (HuNOS2tg/mNos2^−/−^) mice, termed HN).

This mutation helps address differences between the human and mouse innate imune responses, where human macrophages, express little NOS2, and generate much less nitric oxide (NO) in response to inflammatory stimuli, compared to mouse macrophages [17]. Introducing the human NOS2 gene lowers the amounts of NO produced, to help render the innate immune system more human like by bringing the mouse immune/redox activity more in tune with the human [18].

Mice were fed a control diet for their whole life (2001 Lab Diet; denoted Ctrl), or swithced to a high fat diet for the last 4 months before sacrifice (D12451i, Research Diets; denoted HFD). A total of 169 mice were scanned using diffusion weighted MRI (dMRI). See Table 1 for more details.

**Table 1:** Description of the mice population. For each genotype, some mice were given high fat diet (HFD) and some had the humanized NOS2 gene.

Genotype	N	Sex (Female)	Age (Mean; Std)	High Fat Diet	Immunity
ApoE 2	58	30	16.12; 1.8	15	25
ApoE 3	54	27	15.25; 1.54	25	20
ApoE 4	57	28	15.84; 1.76	15	30

Specimens were prepared for imaging as described in [19]. Briefly mice were anesthetized with ketamine/xylazine (100 mg/kg ketamine, 10 mg/kg xylazine) and perfused via the left cardiac ventricle. Blood was cleared with 0.9

### 2.2 Image collection and preprocessing

Mouse brain specimens were imaged at 9.4 T, as described in [20], using a 3D spin echo diffusion weighted imaging (SE DWI) sequence with TR/TE: 100 ms/14.2 ms; matrix: 420 × 256 × 256; FOV: 18.9 × 11.5 × 11.5 mm, BW 62.5 kHz; reconstructed at 45 *μ* m isotropic resolution. Diffusion weighting was applied along 46 directions, using 2 diffusion shells (23 directions using a b value of 2,000 s/*mm*^2^ and 23 directions using a b value of 4,000 s/*mm*^2^); we also acquired 5 non-diffusion weighted images (b0). The max diffusion pulse amplitude was 130.57 Gauss/cm; duration 4 ms; separation 6 ms. We used eightfold compressed-sensing acceleration and reconstructed images using BART [21,22].

Diffusion tensor properties (i.e., fractional anisotropy, orientation distribution functions, and tractograms) were reconstructed via multishell, multi-tissue constrained spherical deconvolution (CSD) [23] and filtered using Spherical-deconvolution Informed Filtering (SIFT) [24] with MRtrix3 [25], producing around two million tracts per brain. To produce regional estimates of volume and FA we have used pipelines implemented in a high-performance computing environment for image reconstruction [26], and atlas based segmentation [27,28] using a symmetrized mouse brain atlas [29,30] with 332 regions, 166 for each hemisphere.

### 2.3 Single subject networks and covariates

Each mouse brain image was segmented into 332 brain regions using SAMBA and the symmetrized mouse brain atlas previously used for connectomic analyses [27,28]. Diffusion (FA) improves contrast, increasing the accuracy segmentations in our experience, at least with models of risk, and in the absence of demyelination or lesions [29]. In our preclinical mouse brain images we use a high resolution, 45 *μ* u isotropic resolution that has been shown to provide sensitive connectomes for detecting the impact of AD risk factors [29]. The volume of each brain region was computed by counting the number of voxels in each region and multiplying this by the voxel size, and the average fractional anisotropy (FA) for each brain region was calculated using MRtrix3 [25] (see Sects 2.1 and 2.2 for more details).

**Fig 2:**
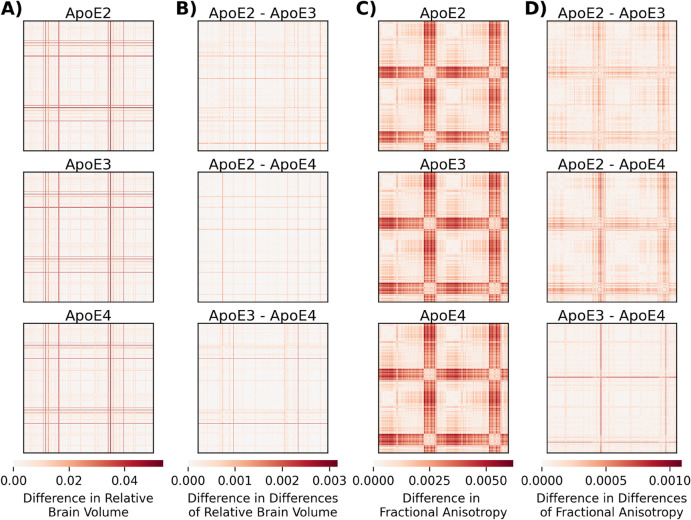
Visualization of the Distance Matrices from Brain Volume (BV) and Fractional Anisotropy (FA) Measurements. **(A)** The averaged difference of **brain volumes** between all pairs of brain regions. Each row represents one of the three ApoE genotypes. **(B)** The difference in differences of relative brain volume between each possible pairs of three ApoE genotypes. **(C)** The averaged difference of **fractional anisotropy** between all pairs of brain regions. Each row represents one of the three ApoE genotypes. **(D)** The difference in differences of fractional anisotropy between each possible pairs of three ApoE genotypes.

To compute single-subject networks, we first normalized brain volume and FA by dividing by the total brain volume and maximum FA, respectively, then computed the absolute difference between all pairs of brain regions, resulting in a distance matrix of size 332×332, where each element of the matrix corresponds to the difference of either brain volume or FA from a pair of brain regions. This resulted in two networks per mouse, one from each of the two data modalities. For all subsequent analyses, we treated these networks as undirected (since we computed absolute differences), weighted so that all values lie between 0 and 1, and loopless (e.g. there is no difference in volume of a particular region to itself). Fig 2A and 2C shows the averaged distance matrices for brain volume and FA, respectively. To better understand the distance matrices, we then computed the difference of the distance matrices from pairs of genotypes, which highlights qualitative differences in the distance matrices (Fig 2B and 2D).

Besides brain volume and fractional anisotropy (FA) data, we collected additional information for each mouse that might predict Alzheimer’s disease risk. This included: genotype (ApoE2, ApoE3, or ApoE4, which have varying risk levels), sex (female or male, as females are believed to be more susceptible), age (categorized as below or above the median age for each genotype, since older age is a major risk factor for late-onset AD), immunity (presence of the humanized NOS2 gene, denoted as HN, for a more human-like immune system), and diet (control or high-fat diet, denoted as HFD, with HFD expected to increase vulnerability).

Next, we present a model for multiple networks and methods for obtaining new representations of all the networks, which takes into account the inherent structure and dependencies within networks.

### 2.4 Statistical modeling of networks

Statistical models for networks allows us to model all of the inherent dependencies across vertices (brain ROIs) in networks, and transforms the data in which we can apply traditional statistical and machine learning tools that would otherwise be inappropriate for network data. The joint random dot product graph (jrdpg) model provides a way to model weighted networks and allow us to obtain a Euclidean representation of networks using statistically principled procedures [31–33]. In this model, a vertex is a region-of-interest (ROI) in the brain, which is represented as a low-dimensional vector called a latent position. The probability of one ROI connecting to another is determined by the dot product of the corresponding latent positions. In other words, a matrix containing the latent positions of all ROIs is a representation of the underlying distribution of the networks.

Given our networks derived from brain volume and FA data, we can obtain the low-dimensional latent positions using a joint embedding technique called omnibus embedding (omni) (see Sect 2.5 for more details); that is, embed all of the networks from derived from brain volume and then embed networks from the FA independently. We will then concatenate the output embeddings for each modality giving us a final feature set that will be used for subsequent analysis. The dimensionality of the latent position is determined by an automatic elbow detection algorithm [34], which is 3 for both brain volume and FA. We finally obtain a 6 dimensional latent position vector per brain region, resulting in a matrix of size 332×6 per mouse, which we will use for our subsequent analysis. Next, we detail a framework for understanding and quantifying the differences in these maps and how we construct a hypothesis test using latent positions and predictor variables.

### 2.5 Graph theory preliminaries

Networks (or graphs) are convenient mathematical objects for representing connectomes. A network *G* consists of the ordered pair (*V*,*E*), where *V* is the set of vertices and *E* is the set of edges. The set of vertices can be represented as V={1,2,…,n}, where |V|=n is the number of vertices. The set of edges is a subset of all possible connections between vertices (i.e., E⊆ V × V). We say tuple (i,j)∈E if there exists an connection between vertex *i* and vertex *j*. In many connectomics datasets, edges have associated edge weights: these are real-valued numbers that encode quantitative information about a connection between two vertices.

### 2.6 Statistical models

Statistical modeling of connectomics data enables the principled analysis of these high-dimensional, network-valued data. Random network models treat individual connectomes as random variables, enabling mathematical characterization of network structure and accounting for noise within and across observed samples. Treating connectomes as random network-valued variables sampled from these random network models enables the formulation of hypothesis tests that can be used to identify connective differences at multiple levels across numerous phenotypic profiles. For a thorough discussion of statistical models for network-valued data, see [35,36].

#### 2.6.1 Random dot product graph model.

The Random Dot Product Graph (rdpg) is a type of independent edge model. In this model, each element of an adjacency matrix is sampled independently from a Bernoulli distribution:


$${𝐀}_{ij}\sim \text{Bernoulli}({𝐏}_{ij})$$

Given the number of vertices *n*, the matrix 𝐏 is a *n* × *n* matrix of edge-wise connection probabilities with elements in [0,1]. We can construct various models depending on the constraints imposed on 𝐏. Note that we assume that 𝐏 has no self-loops (i.e. $\text{diag}(𝐏)=\vec{0}$) and is undirected (i.e. ${𝐏}^{⊤}=𝐏$).

In the random dot product graph (rdpg), the probability of a connection ${𝐏}_{ij}$ between two vertices *i* and *j* is determined by the vertices. Each vertex i∈V is associated with a low-dimensional *latent position* vector, ${𝐗}_{i}$, in the Euclidean space ${\mathbb{R}}^{d}$. The probability of connection between vertices *i* and *j* is given by the dot product (i.e ${𝐏}_{ij}={𝐗}_{i}{𝐗}_{j}^{⊤}$). Thus, in a *d*-dimensional rdpg with *n* vertices, the rows of the matrix $𝐗\in {\mathbb{R}}^{n\times n}$ are the latent positions of each vertex, and the matrix of edge-wise connection probabilities is given by $𝐏=𝐗{𝐗}^{⊤}$. Each element of the adjacency matrix is then independently modelled as:


$${𝐀}_{ij}=\text{Bernoulli}({𝐗}_{i}{𝐗}_{j}^{⊤})$$

where ${𝐗}_{i}$ and ${𝐗}_{j}$ are latent positions for vertices *i* and *j*, respectively. rdpg s are highly flexible random network models, in that they can characterize any random network with positive semi-definite structure (e.g., certain types of block models, which are commonly used to model brain networks [35]).

We acknowledge that the original intention of rdpg is to model binary networks, although the model can be naturally extended to handle weighted networks. However, the weighted rdpg models are not well studied, and as such does not enjoy the same statistical guarantees. In the subsequent section, we present an algorithm for estimating latent positions from observed data and describe methods for preprocessing the data to enable interpretation of results within the context of binary networks while still utilizing weighted network data.

### 2.7 Adjacency spectral embedding

The modeling assumptions of rdpg make the estimation of latent positions, which are usually unobserved in practice, analytically tractable. The estimation procedure we use is *adjacency spectral embedding* (ase) [37]. The ase of an adjacency matrix 𝐀 in *d* dimensions is $\hat{𝐗}=\hat{𝐔}|\hat{𝐒}{|}^{1/2}$, where $\left|\hat{𝐒}\right|$ is a diagonal *d* × *d* matrix containing on the main diagonal the absolute value of the top-*d* eigenvalues of 𝐀 in magnitude, in decreasing order, and $\hat{𝐔}$ is an *n* × *d* matrix containing the corresponding orthonormal eigenvectors. This simple and computationally efficient approach results in consistent estimates $\hat{𝐗}$ of the true latent positions 𝐗 [37–39]. The ase depends on a parameter *d* that corresponds to the rank of the expected adjacency matrix conditional on the latent positions; in practice, we estimate this dimension, $\hat{d}$, via the scree plot of the eigenvalues of the adjacency matrix which can be done automatically via a likelihood profile approach [34].

#### 2.7.1 Joint Random Dot Product Graphs (JRDPG).

The jrdpg extends the concept of the rdpg to a collection of *m* random networks all with the same generating latent positions. Similar to a rdpg, given an appropriately constrained Euclidean subspace ${\mathbb{R}}^{d}$, the model is parameterized by a latent positions matrix $𝐗\in {\mathbb{R}}^{n\times d}$ where d≪n. The model is $\left({𝐀}^{\left(1\right)},{𝐀}^{\left(2\right)},\ldots ,{𝐀}^{\left(m\right)}\right)\sim \text{jrdpg}(𝐗)$ where ${𝐀}_{ij}^{\left(l\right)}\sim \text{Bernoulli}({𝐗}_{i}{𝐗}_{j}^{⊤})$ for all i,j∈[n] and l∈[m]. Each network has marginal distribution ${𝐀}^{\left(l\right)}\sim \text{rdpg}(𝐗)$ for all l∈[m], and the matrices ${𝐀}^{\left(1\right)},\ldots ,{𝐀}^{\left(m\right)}$ are conditionally independent given 𝐗 [40,41]. Under the JRDPG model, intuitively, networks of a given modality (volume or FA) have the same properties (they are equal in distribution) across all *m* mice under study (regardless of other factors; e.g., ApoE allele).

#### 2.7.2 Omnibus embedding.

Assume we have *m* observed networks ${𝒢}^{\left(1\right)},{𝒢}^{\left(2\right)},\ldots ,{𝒢}^{\left(m\right)}$ and their associated adjacency matrices, ${𝐀}^{\left(1\right)},{𝐀}^{\left(2\right)},\ldots ,{𝐀}^{\left(m\right)}\in {\mathbb{R}}^{n\times n}$ with *n* vertices that are identical and shared across all networks. If we wish to make comparisons across these networks and assume the networks are rdpg, a naive first-pass approach would be to estimate latent positions via ase, and then compare these estimated latent positions. This approach, however, runs into the issue that latent positions estimated via spectral methods are rotationally non-identifiable, in that two unique latent position matrices may be equally valid for a given network provided the latent position matrices are rotations of one another (i.e., they will be unequal in Frobenius norm). This makes comparisons difficult, as latent positions estimated separately via ase will rarely be rotationally oriented with respect to one another. This motivates the omni embedding for multiple network models. To begin, we first construct the omnibus matrix **O**, which is the mn×mn matrix whose n×n sub-matrices are the entries:


$$𝐎=[\begin{array}{cccc} {𝐀}^{\left(1\right)} & \frac{1}{2}\left({𝐀}^{\left(1\right)}+{𝐀}^{\left(2\right)}\right) & \cdots & \frac{1}{2}\left({𝐀}^{\left(1\right)}+{𝐀}^{\left(m\right)}\right)\\ \frac{1}{2}\left({𝐀}^{\left(2\right)}+{𝐀}^{\left(2\right)}\right) & {𝐀}^{\left(2\right)} & \cdots & \left({𝐀}^{\left(2\right)}+{𝐀}^{\left(m\right)}\right)\\ \vdots & \vdots & \ddots & \vdots \\ \frac{1}{2}\left({𝐀}^{\left(m\right)}+{𝐀}^{\left(1\right)}\right) & \frac{1}{2}\left({𝐀}^{\left(m\right)}+{𝐀}^{\left(2\right)}\right) & \cdots & {𝐀}^{\left(m\right)} \end{array}]$$

We then embed the omnibus matrix using ase as:


$$\hat{𝐙}=\text{ase}(𝐎)=[\begin{array}{c} {\hat{𝐗}}^{\left(1\right)}\\ {\hat{𝐗}}^{\left(2\right)}\\ \vdots \\ {\hat{𝐗}}^{\left(m\right)} \end{array}]\in {\mathbb{R}}^{mn\times d}$$

Where *d* is the latent dimensionality. Collectively, this procedure is known as the omnibus embedding (omni). Each n×d submatrix ${\hat{𝐗}}^{\left(i\right)}$ denotes the estimated latent position matrix of a given modality (FA or volume) for the $i^{\text{th}}$ mouse. Under the jrdpg model (e.g., the networks for a given modality across all *m* mice are equal in distribution), the resulting estimated latent positions will be similar (i.e., in Frobenius norm) across all networks; conversely, if the networks for a given modality across all *m* mice are *not* equal in distribution (e.g., if there are discernable differences across certain groups of mice; e.g., ApoE allele), the resulting estimated latent positions are different [35,42]. This motivates the use of latent positions estimated via omni for subsequent statistical hypothesis testing frameworks. Here, the latent dimensionality *d* is chosen as the second elbow from the scree plot of the spectral embedding of **O**. These elbows are estimated automatically via analysis using the profile log-likelihood [43].

### 2.8 Hypothesis testing for discovering vulnerable regions

We sought to understand whether these single-subject maps differ in order to identify and characterize how certain predictor variables, such as ApoE genotypes, affect brain regions. A simple approach is to ask whether the brain volume and FA of a region are significantly different across a predictor. One way to formalize the question into a hypothesis test is as a *K*-sample testing problem. Let ${\text{y}}^{\left(\text{i}\right)}$ represent measurements (in this case, latent positions estimated via omni for the FA and volume networks) for i=1,…,m samples. For each sample, we have additional covariates ${\text{t}}^{\left(\text{i}\right)}$ which denote membership into one-of-*K* groups. For example, ${\text{t}}^{\left(\text{i}\right)}$ can denote different genotypes such as ApoE2, ApoE3, and ApoE4, and ${\text{y}}^{\left(\text{i}\right)}$ can be measurements, such as estimated latent positions across brain volume and FA. Assume that if *t*^(*i*)^ = *k*, that measurements ${\text{y}}^{\left(\text{i}\right)}$ are sampled independently and identically from some distribution *F*_*k*_. A reasonable test would be whether the measurements differ depending on group; this can be formalized as the *K*-sample test:

$$H_{0}:F_{1}=F_{2}=\cdots =F_{K},\;H_{A}:\exists \;k\neq l\text{ s.t. }F_{k}\neq F_{l}$$ (1)

In words, under the null hypothesis, the measurements do not differ across groups; under the alternative, there exists at least one pair of groups for which the measurement distributions are unequal. Intuitively, rejection of the null hypothesis in favor of the alternative has the interpretation that the grouping encodes meaningful differences in the measurements for a particular region (the region is vulnerable). This test can be generalized to the case where there are multiple predictors, or multi-way *K*-sample testing. For example, suppose that *K* denotes our three ApoE genotypes and that for each sample we obtain additional predictor data, such as age and sex. Traditionally, analysis of variance (ANOVA) [44] or multivariate ANOVA (MANOVA) [45] are conventional choices for *k*-sample tests. For multi-way *K*-sample testing, *K*-way ANOVA [46] or *K*-way MANOVA are common choices. However, these tests often do not perform well for high-dimensional or non-Gaussian data, which is typically the case for network data, because their performance depends on assumptions that are generally not present in real data [47,48]. Several non-parametric alternatives have been developed to address this issue; we choose distance correlation (dcorr) for testing [49]. While dcorr is an independence test, the connections between the independence test and the multi-way *K*-sample test, and its advantages in comparison to alternative choices such as *K*-way MANOVA, are detailed in [12]. In particular, *K*-way MANOVA is are typically designed for testing only mean differences, and lack sensitivity to other types of distributional differences (e.g., differences in variance across the *K* groups), and require prescription of inter-correlations that may exist between dimensions of multivariate data such as that we are interested with here. On the other hand, dcorr-based approaches do not feature these limitations [12].

**Hypothesis testing rationale.** Our procedures can be described intuitively as follows: under the null hypothesis (there is no difference in measurements across groups; e.g., Eq (1)), the jrdpg model is flexible for homogeneous networks, the omni embedding provides an effective strategy for estimating the latent positions of these networks, and these estimated latent positions will have approximately the same distribution across groups. Under the alternative hypothesis, the latent positions estimated by omni will differ; e.g., the estimated latent positions will not have the same distribution across groups. We exploit these observations to motivate the use of the estimated latent positions from volume and FA-derived difference matrices for our testing procedures described herein.

For our statistical analyses, we use the estimated latent positions for each ROI jointly across both volume and average FA to test if they differ significantly given some combination of groups (e.g. certain labels). For example, we may test of there is a difference among the ApoE 2, 3, 4 genotypes while considering both sex (male and female), as well as immunity (presence or absence of HN). We then run subsequent analysis comparing subgroups. For example, instead of examining all three genotypes, we can compare ApoE2 (protective of Alzheimer’s in human populations) with ApoE3 and 4 (neutral and a known risk factor for Alzheimer’s in human populations, respectively) while considering gender and alleles, to identify the areas of the brain that are differentially expressed by particular genotypes across particular factors.

We divided the analysis into two distinct categories due to the absence of observed data for certain combinations of factors. For instance, not all mice with a non-HN immunity gene were subjected to a high-fat diet. Consequently, we conducted two separate analyses to understand the impact of human immunity and diet. The first analysis examined the effects of genotypes, sex, age, and immunity (HN and non-HN), while the second analysis explored the effects of genotypes, sex, age, and diet (control and HFD). Specifically, only mice that were given normal diet were considered in the first analysis, and only mice with HN immunity were considered in the second analysis.

### 2.9 *p*-Values and multiple hypothesis correction

In the context of this investigation, effect sizes are measured using the distance correlation (dcorr, [50]). Approaches for performing null hypothesis significance tests via dcorr have traditionally leveraged large numbers of permutations for each test. This can be computationally prohibitive when large numbers of tests are performed, such as those proposed herein. To overcome this hurdle, we leverage recent advances in estimating the null distribution of the dcorr statistic via chi-squared approximation for fast computations [51]. In all figures and tables associated with this work, we are interested in interpreting individual statistical tests at a given significance level. Therefore, we control the familywise error rate (FWER) with the Holm-Bonferroni correction [52].

### Ethics statement

All animal procedures adhered to the Office of Animal Welfare Assurance (OAWA) and Institutional Animal Care and Use Committee (IACUC) guidelines,under protocol A022-21-01, to ensure ethical compliance. Animals were euthanized following American Veterinary Medical Association (AVMA) guidelines, to prepare specimens for imaging as described in [19]. Briefly mice were brought to surgical plane anesthesia with ketamine/xylazine (100 mg/kg ketamine, 10 mg/kg xylazine) and once unresponsive to toe pinch reflex, were perfused via the left cardiac ventricle with 0.9

## 3 Results

### 3.1 Visualizations of distance matrices show qualitative differences across genotypes and imaging features

Distance matrices derived from volume and fractional anisotropy (FA) show distinct patterns across ApoE genotypes (Fig 2). Genotype-related differences are more extensive in FA-derived networks than volume-derived networks.

### 3.2 Effects of risk factors in mice fed a control diet

We performed multi-way hypothesis testing (distance correlation, as-per Sect 2.8) to identify brain regions where structural covariance network patterns (derived from volume and FA) differed significantly across different combinations of risk factors, including ApoE genotypes, sex, age, and immune status.

**Fig 3:**
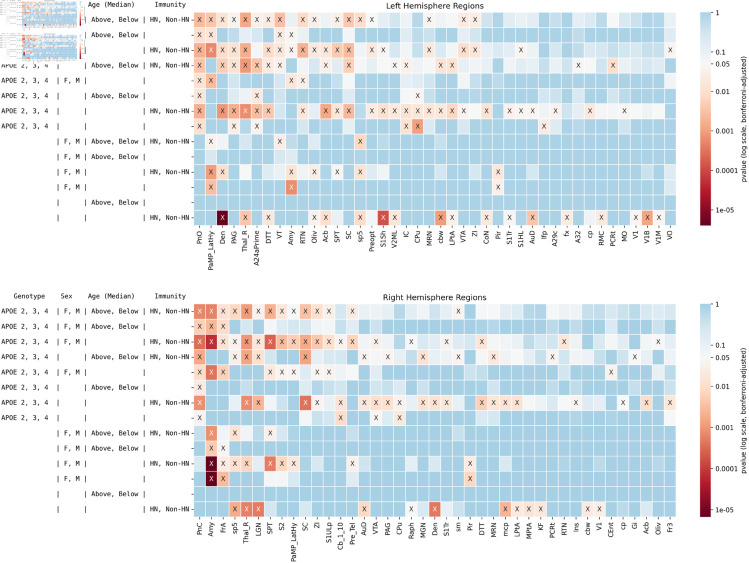
Effect on structural covariance networks of ApoE genotype, sex, age, and innate immunity. The results of tests conducted on each hemisphere are shown on top panel for left hemisphere and bottom panel for the right hemisphere. Significant results are shown by X. Brain regions in which no tests were significant are not shown for visualization purposes. A total of *n* = 114 mice were included in the tests. In all, 207 combinations of genotypes and factors were tested. Effect sizes are found in S1 Figure.

This analysis included only mice fed a control diet (*n*=114), excluding high-fat diet mice, as only mice with the non-humanized ApoE gene were administered the high fat diet. Fig 3 shows the statistical significance of different combinations of factors in various areas of the brain, which are summarized below. Effect sizes for the corresponding factors are given in S1 Figure.

1. Age: median age showed no significant differences.
2. Sex: Significant sex-related differences were notable in regions traditionally associated with hormonal influence, such as the bilateral amygdala and left lateral hypothalamus; also the bilateral piriform cortex; right frontal association cortex. Three regions showed differences in each hemisphere.
3. ApoE Genotype: The genotype factor showed a diverse influence on brain regions including the bilateral caudate putamen (CPu); left pons (PnO, oral part), periaqueductal gray (PAG), cingulate cortex area 24a (A24a), inferior colliculus (IC), and longitudinal fasciculus of pons (lfp); right pons (PnC, caudal part), ventral tegmental area (VTA), and cerebellar nuclei. 6 regions in the left hemipshere and 4 in the right were affected.
4. Immunity: The contrast between mouse and humanized immune system mice yielded the largest number of significant results, including the bilateral dentate nucleus of the cerebellum, thalamus, parietal association cortex, auditory and visual cortex (V1B, V1 motor); left cochlear nucleus, fornix, cerebellar white matter, dorsal tenia tecta; and right raphe nuclei. 18 regions in the left hemisphere and 12 in the right were affected.

The largest number of regions found to differ significantly was for immunity (18 on the left, 12 on the right hemisphere), followed by ApoE allele (6 left, 4 on the right hemisphere), and then sex (3 regions in each hemisphere). We identified a prominent role for the right amygdala and thalamus, as these regions were highly significant for 8 of the 15 comparisons for all factors. Interestingly, areas of the pons are significant in 8 comparisons for each hemisphere, with the left primarily implicating the PnO, and the right the PnC. The interaction of ApoE and immunity revealed a large number of regions (26 left hemisphere, 21 in the right hemisphere); as well as the interaction of ApoE, sex, and immunity (20 in each hemisphere). The combination of all factors revealed 17 regions in the left, and 14 in the right hemisphere – including the bilateral amygdala, thalamus and superior colliculus – along with the right septum and left dentatecerebellar nucleus.

**Fig 4:**
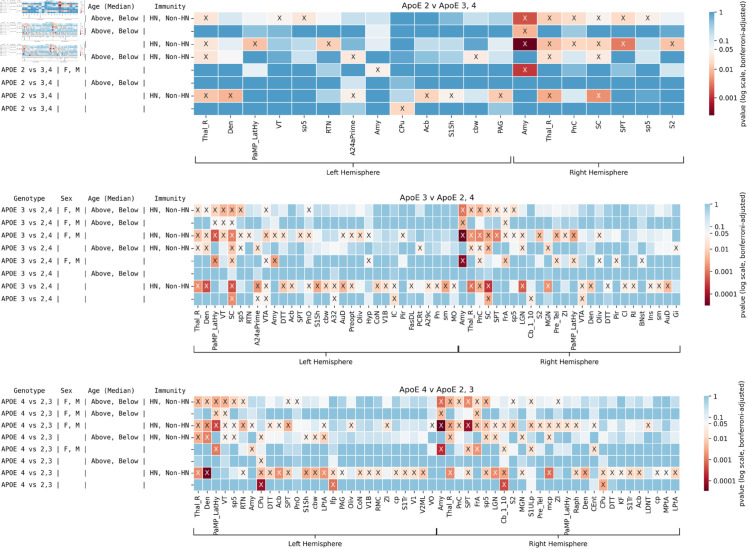
Brain regions showing significant differences in structural covariance patterns for each ApoE genotype versus the other two genotypes, across sex, age, and immune status. The results of tests conducted on different genotypes are shown in each panel. Significant results are shown by X. Brain regions in which no tests were significant are not shown for visualization purposes. A total of *n* = 114 mice were included in the tests. Effect sizes are found in S2 Figure.

We then conducted pairwise comparisons of each ApoE genotype versus the other two, in Fig 4 (effect sizes are given in S2 Figure).

When comparing ApoE2 versus ApoE3 or 4 mice, fewer regions were significantly different; however, we noted a role for the caudate putamen for genotype comparisons; and for the amygdala when examining the role of sex, and the interaction of sex with immunity, where additional regions that played a role included the septum, S2, hypothalamus, and thalamus including its reticular nucleus. Amygdala was present in the interaction of all factors, as well as septum, superior colliculus, and thalamus.

When comparing ApoE3 mice against other strains, we found a role for the superior colliculus, cingulate cortex, ventral tegmental area, and cerebellar nuclei for genotype comparisons. The largest differences in terms of the number of regions appeared for the effect of immunity (34 regions). The interaction of all factors included the thalamus, superior colliculus, sp5, bilateral pons, amygdala, septum, frontal association cortex, VTA, lateral hypothalamus, and dentate nucleus of cerebellum. Sex differences included the amygdala, hypothalamus, bed nucleus of the stria terminalis, and frontal association cortex.

The caudate putamen, the cerebellum, and the longitudinal fasciculus of the pons showed significance when comparing ApoE4 mice against other strains. Age was important within the ApoE4 genotype, and indicated a role for the caudate putamen. 36 regions were affected by immunity. For the interaction of all factors we noted the thalamus rest, septum, pons, and spinal trigemnial nerve bilaterally; additionally, hemispheric locality for the frontal association cortex, S1, S2, thalamic nuclei (reticular, ventral and zona incerta), lateral hypothalamus, amygdala, S1 and S2. Interestingly, sex differences were associated with the entorhinal cortex.

In summary, certain regions that are typically implicated in Alzheimer’s disease, such as amygdala and thalamus, showed significant differences across many of the tests. The effect of immunity in terms of the number of significant regions increased from ApoE2 vs 3 or 4 to ApoE3 vs 2 or 4 to ApoE4 vs 2 or 3.

### 3.3 Effects of high-fat diet on structural covariance patterns

**Fig 5:**
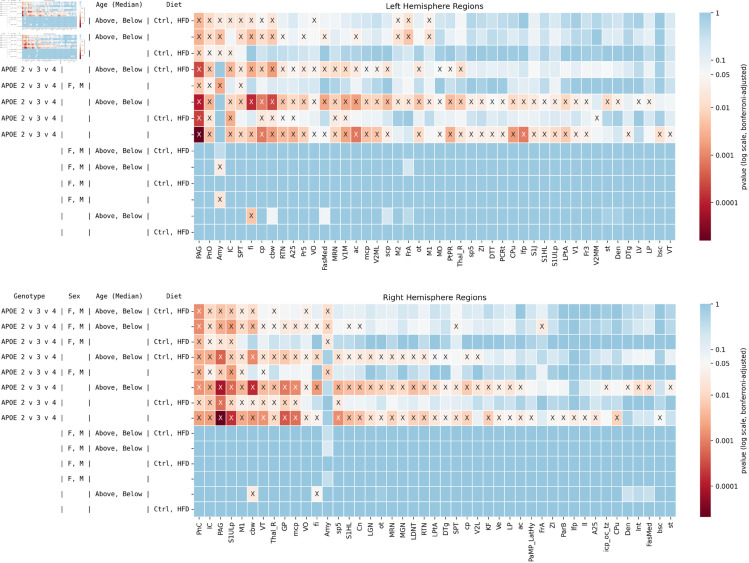
Effect on structural covariance networks of ApoE genotype, sex, age, and diet. The results of tests conducted on each hemisphere are shown on top panel for left hemisphere and bottom panel for the right hemisphere. Significant results are shown by X. Brain regions in which no tests were significant are not shown for visualization purposes. A total of *n* = 75 mice were included in the tests. In all, 154 combinations of genotypes and factors were tested. Effect sizes are found in S3 Figure.

We applied a similar strategy for studying the effects of high-fat diet (HFD) in ApoE mice to better understand the interplay between diet and genotype, along with other factors (age, sex), as shown in Fig 5 (effect sizes in S3 Figure). This analysis included only mice with the humanized immune genotype (HN, *n*=75), because no mice with non-HN were administered HFD.

1. Age: Age showed a significant effect for the fimbria (bilateral), and right cerebellar white matter. Significant interactions were observed with ApoE genotypes for 41 regions in the left hemisphere and 35 regions in the right hemisphere including V1, V2, S1, septum, caudate putamen, cerebellar nuclei, fimbria, and other major white matter tracts, as well as for the lateral ventricles.
2. Sex: Sex only showed a direct effect on one region, the amygdala (left), and showed significant interactions with ApoE genotypes for the bilateral amygdala and periaqueductal gray; left septum; right S1, M1, and inferior colliculus.
3. ApoE genotype: The ApoE genotype factor showed a widespread influence on brain regions, including the bilateral periaqueductal gray (PAG), pons, caudate putamen; right globus palidus, left parietal association cortex, right lateral hypithalamus. Diet interacted with genotype for the bilateral PAG, pons, inferior colliculus, V2, thalamus (ventral); also the left cingulate cortex, and V1; right S1, M1, globus pallidus. The amygdala, M1, inferior colliculus, and PAG were present at the intersection of all factors; as well as left septum and fimbria; and right ventral orbital cortex.
4. Diet: While diet alone did not show significant effects on structural networks, numerous differences were observed in the interaction with genotypes, suggesting that genetic background determines vulnerability to dietary influences. Genotype by diet interaction showed effects in several key regions: on the left pons, inferior colliculus, S1, cingulate cortex (A25), V1 amd V2, and midbrain reticular nuclei, cerebral peduncle and cerebellar white matter; right M1, thalamus, and globus pallidus.

**Fig 6:**
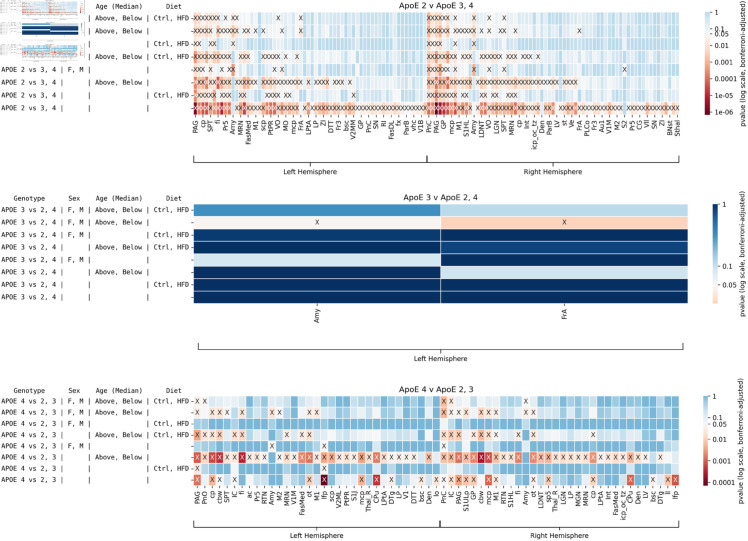
Brain regions showing significant differences in structural covariance patterns for each ApoE genotype versus the other two genotypes, across sex, age, and diet. The results of tests conducted on different pairs of genotypes are shown in each panel. Significant results are shown by X. Brain regions in which no tests were significant are not shown for visualization purposes. A total of *n* = 75 mice were included in the tests. Fewer differences are present between ApoE3 and ApoE2-4 groups than other groupwise comparisons. Interestingly, certain regions that are typically implicated in Alzheimer’s disease, such as the amygdala and thalamus, differ across at least one test for all groupwise comparisons. Effect sizes are found in S4 Figure.

We conducted pairwise comparisons of each ApoE genotype against the other two including dietary comparisons for the HN mice in Fig 6, with effect sizes in S4 Figure. We observe numerous differences in ApoE2 vs 3 or 4 and ApoE4 vs 2 or 3 mice. ApoE3 differed less relative to the other two genotypes and only for genotype by sex by age in the amygdala and frontal association cortex. Genotype by diet showed a role for the PAG and the longitudinal fasciculus of pons for the ApoE4 vs 2-3 comparison, while the ApoE2 vs 3-4 comparison showed differences in 34 regions, including septum.

For ApoE4 vs 2-3 the interaction of genotype by age and diet showed a role for PAG, IC, fimbria, optic tract, cerebral peduncle, cerebellar white matter bilaterally, then unilaterally for midbrain reticular nuclei, M1, V2, pons and white matter tracts including fimbria, cerebral peduncle, optic tracts, spinal trigeminal tract, cerebellar white matter bilaterally.

## 4 Discussion

In this study, we present a methodological framework that integrates macrostructural (volume) and microstructural (FA) brain features into individualized structural covariance networks, enabling multivariate analysis of how multiple risk factors interact to shape brain organization. Unlike traditional approaches that assess one imaging metric or risk factor at a time, our pipeline captures subject-specific network profiles and uses low-dimensional embeddings to identify latent patterns of co-modulation.

Given the complexity of risk factors for LOAD, we have examined jointly changes in volume and FA covariances in aging mice modeling genetic risk for LOAD, as well as the effects of sex, diet and immunity. Our qualitative examination of the average connectomes support an important role of microstructural features such as FA, which may be an important feature to add to the battery of imaging parameters used to predict and follow the course of AD, and in addition to following the more widely studied regional brain atrophy patterns along the lifespan [53].

When studying animals on a regular diet we found that ApoE genotype influenced the network integration of the caudate putamen [54]; the cingulate cortex [55] involved in mediating cognitive influences on emotion, response to threats; ventral tegmental area involved in regulating reward, learning, memory and addiction behaviors [56]; pons whose volume loss has been associated with greater neocortical amyloid burden [57]; periaqueductal gray involved in modulation of pain perception, and its memory, subsequently linked to anxiety and depression [58]; inferior colliculus modulating auditory signal integration, and helping map physical space with both auditory and visual information [59]. These results support that ApoE is involved in modulating brain networks architecture in regions known to be involved in learning, memory and emotion, functions impacted in AD, regions involved in reward processing and psychiatric conditions, such as depression, and with sensory function.

Sex differences consistently pointed primarily to a role of the amygdala, lateral hypothalamus, well known sexually dimorphic structures [60], as well as the piriform cortex, and frontal association cortex. Immunity effects were seen for a large number of regions, including the thalamus, lateral parietal association cortex, auditory and visual cortex, cerebellar white matter and dentate nuclei, but also fornix, the primary pathway of the limbic system.

In terms of common findings for all ApoE genotypes, the amygdala played an important role for sex differences, sex × immunity, sex × age, and for sex × age × immunity; and the thalamus for immunity, age and immunity, sex and immunity and the intersection of all factors.

The specific effect of ApoE2 was important for the caudate putamen (CPu), and for this group, immunity identified 8 regions, pointing to a role for the thalamus bilaterally, as well as superior colliculus, accumbens, S1, cingulate cortex, and dentate cerebellar nucleus, predominantly for the left hemisphere. Sex differences isolated the amygdala. In addition to the amygdala, the interaction between sex and immunity pointed to a role for the septum. Age and immunity pointed to a role for the thalamus, bilaterally, and also cingulate cortex and superior colliculus. The intersection of all risk factors identified changes in 9 regions, underlining the bilateral amygdala, thalamus, and spinal trigeminal tract; and right septum, superior colliculus, pons.

The presence of ApoE3 indicated additional regions (15 total) changed with the combination of risk factors, besides the regions found for ApoE2. These included the frontal association cortex, the lateral hypothalamus, and the dentate cerebellar nuclei. Immunity identified 34 changed regions, including the claustrum and insula.

The presence of ApoE4 identified 17 regions where affected by all risk factors, adding the septum, S1 and S2; while 36 were affected by immunity, including the accumbens, septum S1, V1, V2 and parietal association cortices. Age was associated with changes to the caudate putamen for ApoE4 only. ApoE4 appeared to be most sensitive to interactions for age, sex, and immunity for animals on a regular diet, and in particular for immunity.

When studying animals exposed to control and high fat diet, the different ApoE alleles affected 37 regions, far greater than in the previous analysis focused on only control diet mice (6 and 4 regions in the left/right hemisphere respectively). We observe many significant changes for the periaqueductal gray, which has been previously linked to consummatory behaviors and responses to reward, and has been proposed to mediate behaviors related to food consumption [61], where the bed nucleus of stria terminalis (BNST) and lateral hypothalamic GABAergic projections to the periaqueductal gray (PAG) may regulate feeding [62]. The interaction between genotype and diet showed around 10 regions, including the PAG, pons [63], and inferior colliculus which has been shown to respond to high levels of circulating glucose [64]. Interestingly, the cerebellar white matter, which has been shown to have a high inflammatory response to a high fat diet [65], was also prominent in the interaction of ApoE genotype with age and diet. This interaction showed effects in the fimbria, suggesting alterations in the memory processing system. The interaction of ApoE genotype, sex, and diet revealed a small set of regions including periaqueductal gray (PAG), pons, amygdala and inferior colliculus, primary somatosensory cortex (S1), and fimbria. The interaction of all risk factors included the amygdala, similarly to our previous analyses, but also the PAG, motor cortex, and fimbria.

ApoE genotype-specific changes were more pronounced in ApoE2 and ApoE4 mice than in ApoE3 mice, indicating resilience of ApoE3 mice to network changes following a high fat diet. The ApoE2 effect was present in a large number of regions (>70), including the bed nucleus of stria terminalis (BNST) periaqueductal gray (PAG), and globus pallidus (GP), associated with alcohol and opiate abuse [66]. The interaction with sex affected 11 regions, including the septum and the amygdala. The effect of ApoE4 was present in 25 regions, and the interaction with aging affected 58 regions.

While many of the regions identified (e.g., amygdala, caudate putamen, thalamus) have been previously implicated in Alzheimer’s disease models, our findings provide a more holistic view of how age, sex, genotype, immune activation, and diet jointly influence brain architecture. Importantly, this framework allows for individualized characterization and lays the groundwork for applying multimodal connectomics to behavioral prediction, early risk stratification, and intervention monitoring.

### 4.1 Comparison with status of the field

We believe that our methods advance the field in several important ways:

1. Integration of Volume and Texture Networks: Unlike previous studies that typically examine either brain volume or texture (e.g., fractional anisotropy, FA) in isolation, we jointly analyzed these features using individualized structural covariance networks. This dual-modality approach allowed us to capture more nuanced network-level alterations that may underlie Alzheimer’s Disease (AD) risk.
2. Single-Subject Network Modeling: Most existing work relies on group-level structural covariance networks, limiting sensitivity to individual variability. We introduce individualized network representations for each mouse, leveraging distance correlation for robust multi-factor testing. This methodological advance enables precise identification of brain regions most vulnerable to combinations of risk factors.
3. Multifactorial Interaction Effects: By modeling the interplay of age, sex, ApoE genotype, immune status, and diet, we reveal synergistic effects that were not apparent in single-factor analyses. For example, the amygdala, thalamus, and periaqueductal gray emerged as key regions at the intersection of multiple risk factors—highlighting their role in the early stages of network dysfunction in AD. The involvement of the PAG and amygdala in network dysfunction aligns with early mood and autonomic symptoms in prodromal AD.
4. Discovery of Resilience Patterns: Our finding that ApoE2 mice exhibit fewer age- and diet-related network changes adds to the understanding of genetic resilience. In contrast, ApoE4 mice showed extensive vulnerability, especially under inflammatory and dietary stressors—providing a new framework for how environmental and genetic risk converge on brain network architecture.
5. Novel Biomarkers for Early Detection: By characterizing shifts in both macrostructure and microstructure through network embeddings, we identify potential early biomarkers for AD risk that precede overt cognitive decline or regional atrophy—offering translational potential for preclinical assessment.
6. Complementary Computational Approach: While other integrative methods have employed multicanonical correlation [67–69], similarity-driven multiview embeddings [70], SVM [71], or deep learning models [72–74], our distance correlation-based approach offers a more interpretable alternative that still captures complex, nonlinear relationships in multidimensional datasets. This addresses a critical gap in the current analytical landscape for neurodegenerative disease research, where transparency and interpretability are increasingly valued alongside predictive power.

These contributions not only expand upon current understanding of AD pathophysiology in mouse models but also offer methodological innovations that could be adapted to human neuroimaging studies [68], ultimately supporting network disease propagation models [75,76].

### 4.2 Limitations

While our study provides valuable insights into structural network alterations associated with AD risk factors, several limitations warrant consideration.

A key methodological consideration is our use of absolute pairwise differences between normalized regional values to derive individualized structural networks. This approach captures magnitude of dissimilarity between regions but does not preserve directionality, meaning our latent dimensions reflect patterns of relative dissimilarity across the brain without indicating specific regional increases or decreases. This trade-off enables holistic, multivariate characterization of network reorganization across interacting risk factors, though at the expense of direction-dependent anatomical specificity.

Although we did not assess cognitive outcomes, the structural network changes we identified may represent early alterations preceding behavioral manifestations. The observed interactions between high-fat diet and ApoE genotype likely reflect broader metabolic dysregulation, particularly impaired cholesterol homeostasis [77], rather than AD-specific pathophysiology. Additionally, our mouse models represent risk factors for AD rather than the disease itself [78]. Therefore, these findings should be interpreted as systematic risk mechanisms converging on brain networks, not as direct markers of late-onset Alzheimer’s disease.

Age showed surprisingly uniform effects across brain regions, possibly due to our relatively narrow age range, which may have limited our ability to detect more nuanced age-related interactions with other risk factors. Additional limitations include the restricted analysis of diet effects to the ApoExHN lines with associated reduction in statistical power.

Furthermore, mouse models cannot fully capture the genetic complexity and population heterogeneity seen in human AD [79]. Sex-specific analyses would also benefit from incorporating ovariectomized female mice to better model menopause-associated effects [80], particularly relevant given the higher prevalence of AD in post-menopausal women.

### 4.3 Conclusion

We present the application of novel models that integrate multivariate data to account for changes in multiple imaging based parameters. Our study in mouse models of genetic risk for LOAD revealed a role for the amygdala, caudate putamen, and PAG in normally aging mice, widespread effects of innate immunity and accentuated, genotype specific changes in response to a high fat diet than under control conditions.

## Supporting information

### Effect sizes for statistical tests

S1 Fig Effect sizes on structural covariance networks across ApoE genotype, sex, age, and innate immunity. Analogous to Fig 3, but illustrating the effect sizes (distance correlation) instead of the *p*-value of the statistical tests. Significant results are shown by X.
(TIF)

S2 Fig Effect sizes across brain regions in structural covariance patterns for each ApoE genotype versus the other two genotypes, across sex, age, and immune status. Analogous to Fig 4, but illustrating the effect sizes (distance correlation) instead of the *p*-value of the statistical tests. Significant results are shown by X.
(TIF)

S3 Fig Effect sizes on structural covariance networks across ApoE genotype, sex, age, and diet. Analogous to Fig 5, but illustrating the effect sizes (distance correlation) instead of the *p*-value of the statistical tests. Significant results are shown by X.
(TIF)

S4 Fig Effect sizes across brain regions in structural covariance patterns for each ApoE genotype versus the other two genotypes, across sex, age, and diet. Analogous to Fig 6, but illustrating the effect sizes (distance correlation) instead of the *p*-value of the statistical tests. Significant results are shown by X.
(TIF)
