# Supplementary figures and images for: Network biomarkers of Alzheimer’s disease risk derived from joint volume and texture covariance patterns in mouse models
Source: PLoS One. 2025 Aug 12;20(8):e0327118. doi: 10.1371/journal.pone.0327118 (PMC12342247; doi:10.1371/journal.pone.0327118)

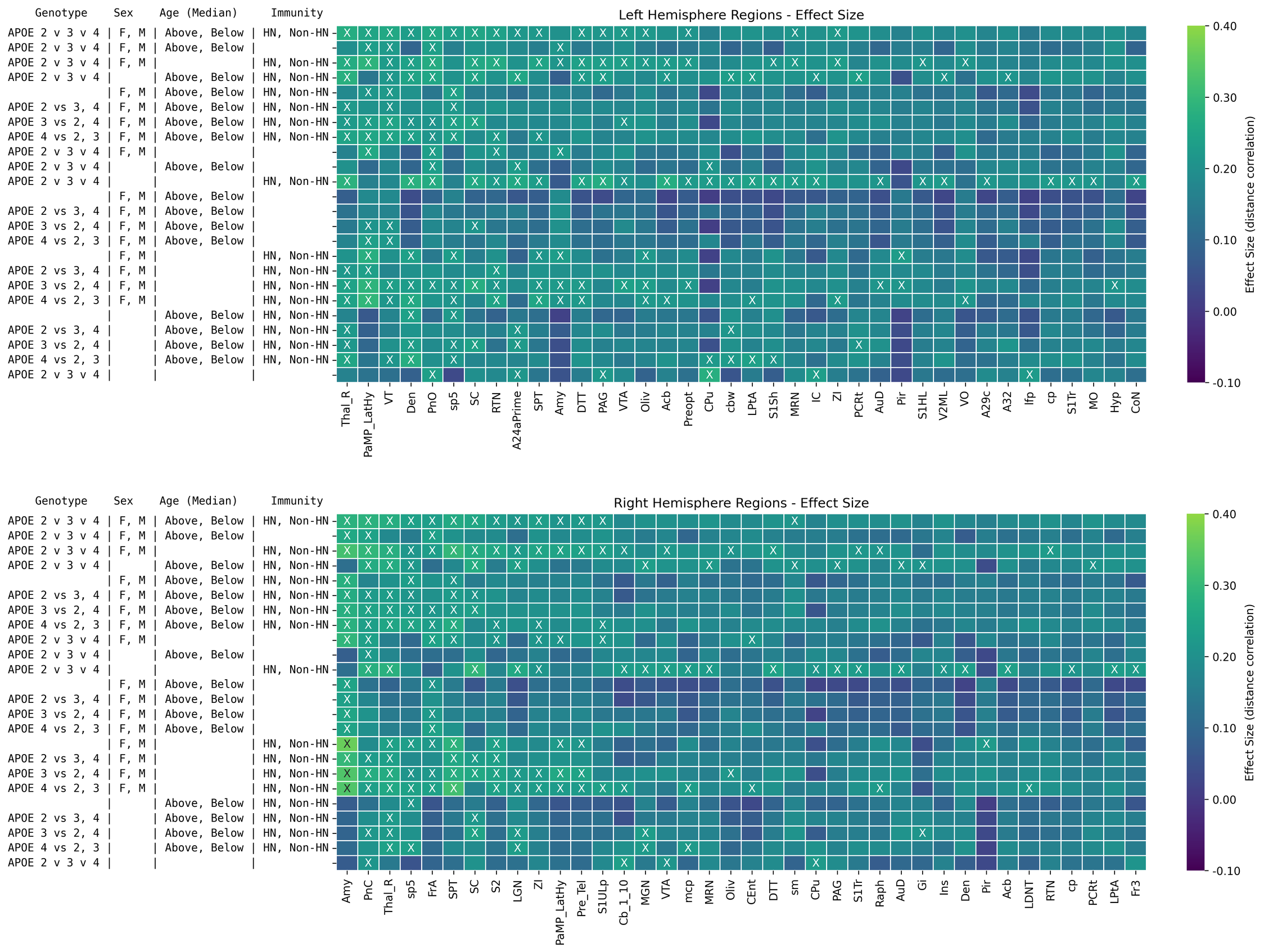

Supplement: S1 Fig — Analogous to Fig 3, but illustrating the effect sizes (distance correlation) instead of the p-value of the statistical tests. Significant results are shown by X. (TIF) [file pone.0327118.s001.tif]

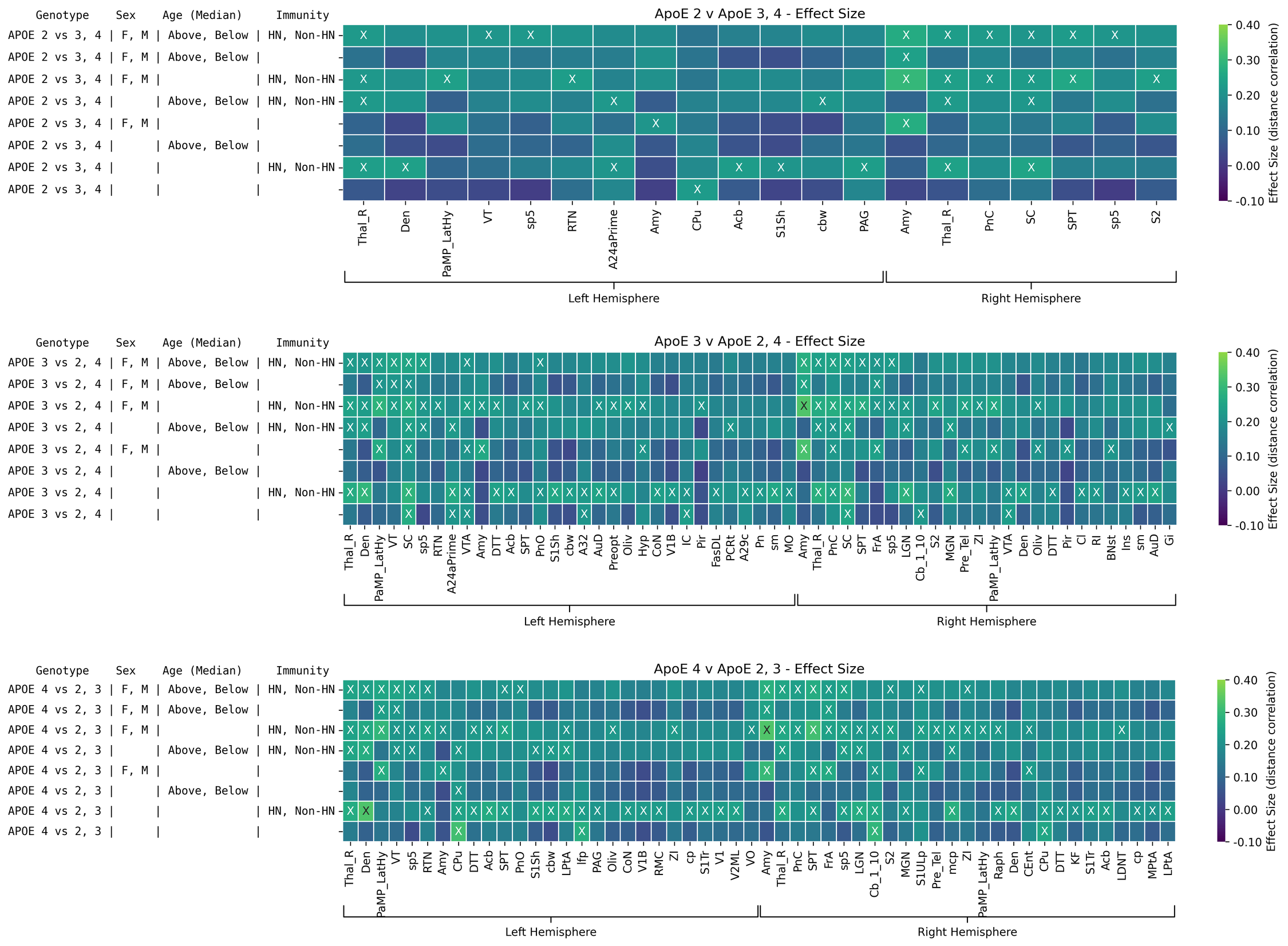

Supplement: S2 Fig — Analogous to Fig 4, but illustrating the effect sizes (distance correlation) instead of the p-value of the statistical tests. Significant results are shown by X. (TIF) [file pone.0327118.s002.tif]

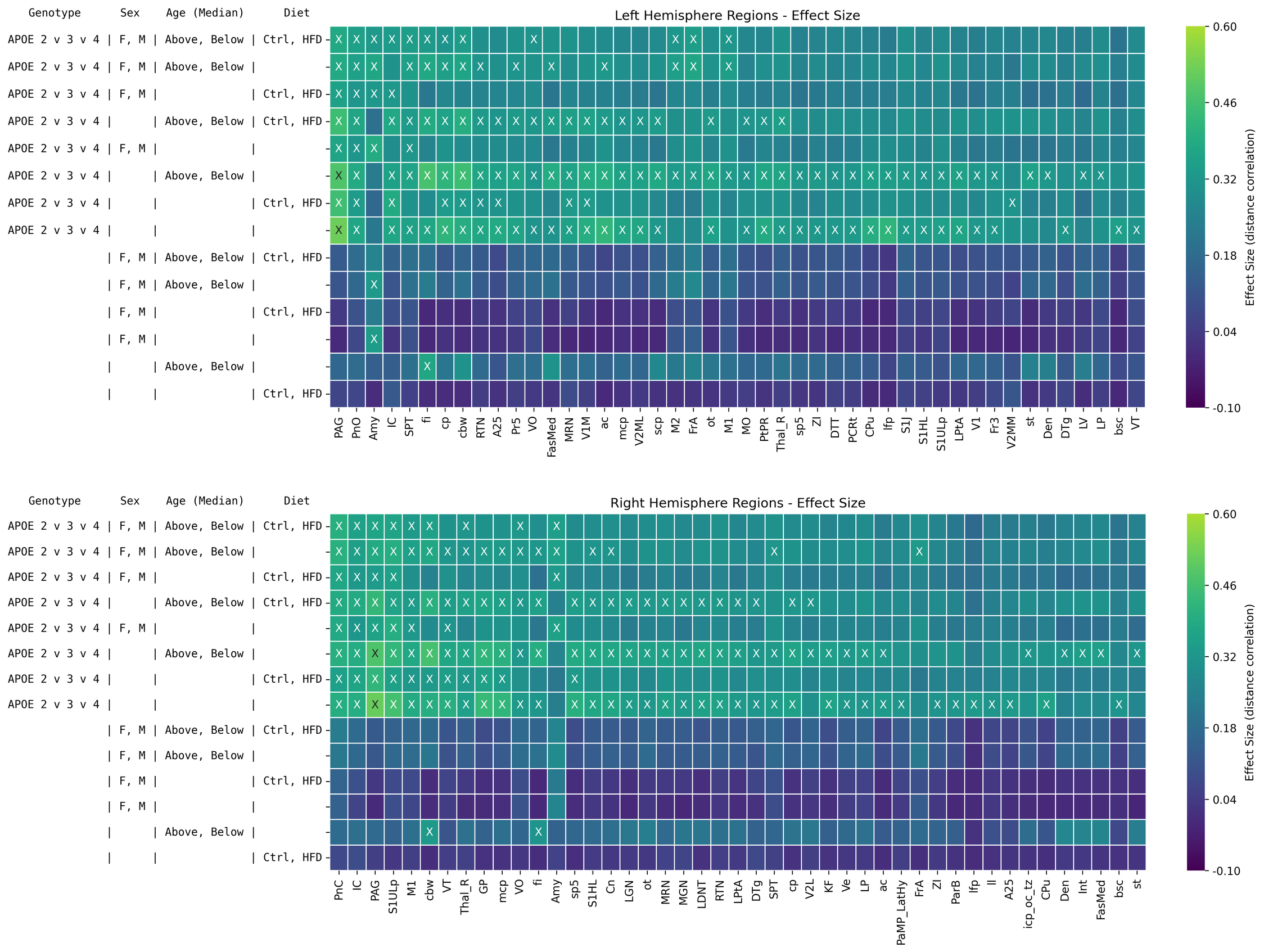

Supplement: S3 Fig — Analogous to Fig 5, but illustrating the effect sizes (distance correlation) instead of the p-value of the statistical tests. Significant results are shown by X. (TIF) [file pone.0327118.s003.tif]

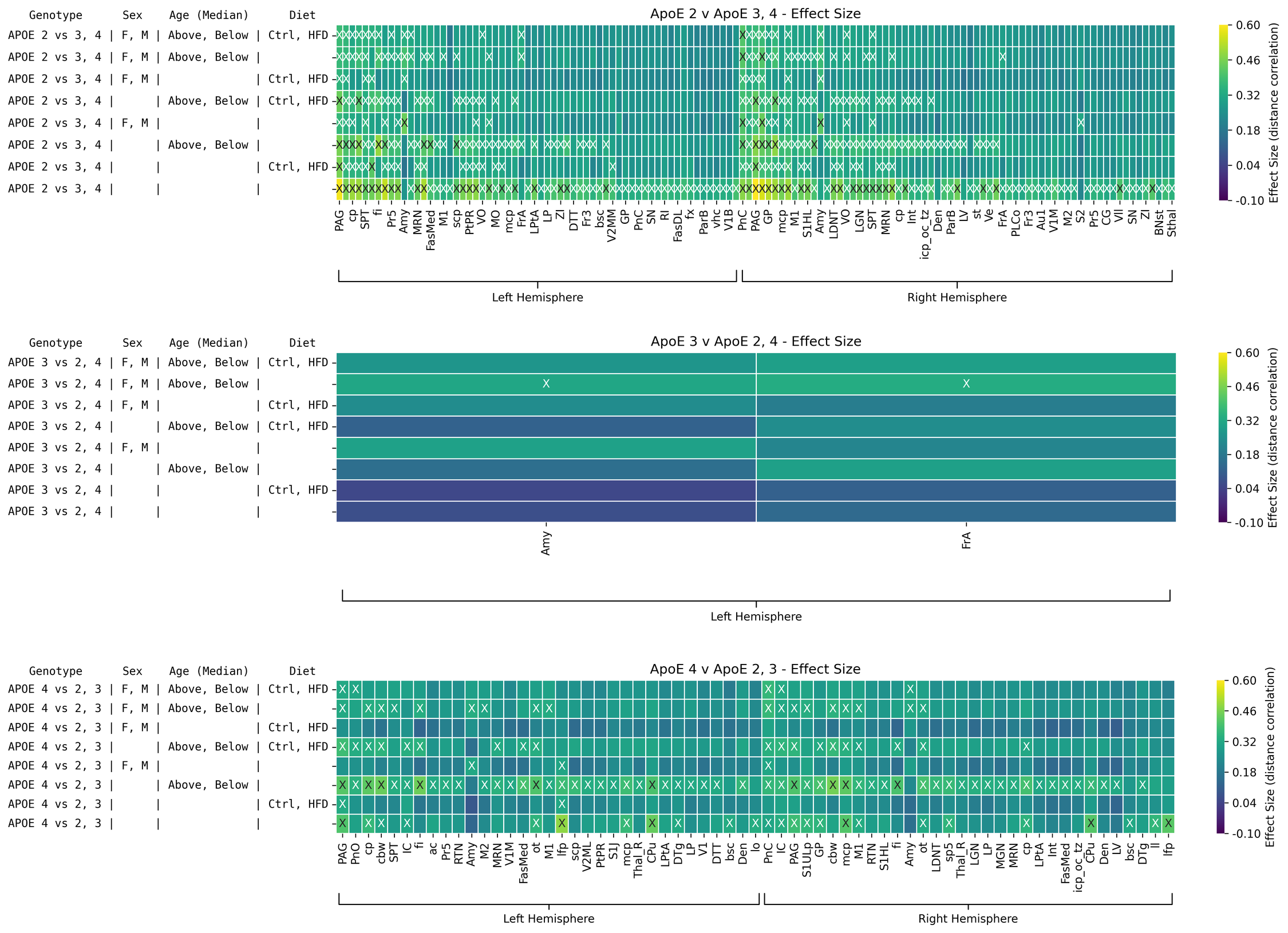

Supplement: S4 Fig — Analogous to Fig 6, but illustrating the effect sizes (distance correlation) instead of the p-value of the statistical tests. Significant results are shown by X. (TIF) [file pone.0327118.s004.tif]
